# The Many Faces of Graphene as Protection Barrier. Performance under Microbial Corrosion and Ni Allergy Conditions

**DOI:** 10.3390/ma10121406

**Published:** 2017-12-08

**Authors:** Carolina Parra, Francisco Montero-Silva, Dana Gentil, Valeria del Campo, Thiago Henrique Rodrigues da Cunha, Ricardo Henríquez, Patricio Häberle, Carolina Garín, Cristian Ramírez, Raúl Fuentes, Marcos Flores, Michael Seeger

**Affiliations:** 1 Laboratorio de Nanobiomateriales, Departamento de Física, Universidad Técnica Federico Santa María, Valparaíso 2390123, Chile; monteroster@gmail.com (F.M.-S.); danagentil5@gmail.com (D.G.); 2 Departamento de Física, Universidad Técnica Federico Santa María, Valparaíso 2390123, Chile; valeria.delcampo@usm.cl (V.d.C.); ricardo.henriquez@usm.cl (R.H.); patricio.haberle@usm.cl (P.H.); 3 Departamento de Física, CTNanotubos, Universidade Federal de Minas Gerais, Belo Horizonte 31310260, Brazil; thyagoh@yahoo.com.br; 4 Instituto de Química, Pontificia Universidad Católica de Valparaíso, Valparaíso 3100000, Chile; carolina.garin@pucv.cl; 5 Departamento de Ingeniería Química y Ambiental, Universidad Técnica Federico Santa María, Valparaíso 2390123, Chile; cristian.ramirez@usm.cl; 6 Departamento de Industrias, Universidad Técnica Federico Santa María, Valparaíso 2390123, Chile; raul.fuentes@usm.cl; 7 Laboratorio de Superficies y Nanomateriales, Departamento de Física, Facultad de Ciencias Físicas y Matemáticas, Universidad de Chile, Santiago 8370448, Chile; mflorescarra@ing.uchile.cl; 8 Departamento de Química, Universidad Técnica Federico Santa María, Valparaíso 2390123, Chile; michael.seeger@usm.cl

**Keywords:** microbial corrosion, CVD, graphene, nickel, sweat

## Abstract

In this work we present a study on the performance of CVD (chemical vapor deposition) graphene coatings grown and transferred on Ni as protection barriers under two scenarios that lead to unwanted metal ion release, microbial corrosion and allergy test conditions. These phenomena have a strong impact in different fields considering nickel (or its alloys) is one of the most widely used metals in industrial and consumer products. Microbial corrosion costs represent fractions of national gross product in different developed countries, whereas Ni allergy is one of the most prevalent allergic conditions in the western world, affecting around 10% of the population. We found that grown graphene coatings act as a protective membrane in biological environments that decreases microbial corrosion of Ni and reduces release of Ni^2+^ ions (source of Ni allergic contact hypersensitivity) when in contact with sweat. This performance seems not to be connected to the strong orbital hybridization that Ni and graphene interface present, indicating electron transfer might not be playing a main role in the robust response of this nanostructured system. The observed protection from biological environment can be understood in terms of graphene impermeability to transfer Ni^2+^ ions, which is enhanced for few layers of graphene grown on Ni. We expect our work will provide a new route for application of graphene as a protection coating for metals in biological environments, where current strategies have shown short-term efficiency and have raised health concerns.

## 1. Introduction

When metals are in contact with biofilms, which naturally form under the exposure of materials to ambient moisture, miscellaneous metabolic activities lead to the production of organic and inorganic acids, volatile compounds and other chemical reactions with the metals. These processes induce a highly accelerated deterioration of the substrate known as microbiologically influenced corrosion or microbial corrosion [1]. This phenomenon represents an enormous burden to maintenance cost in metal-based infrastructure [2,3].

Current strategies to prevent microbial corrosion in metallic structures aim to attack biofilms that cause microbial corrosion. They mainly include physical methods (e.g., flushing) and chemical methods (e.g., biocides) [2,4]. The use of protective epoxy coatings in order to isolate metals from environment and the application of passivation layers (e.g., thiol-based mono-layers) have been considered also as standard mitigation strategies [4]. However, compared to the lifetime of the objects being protected, all these control approaches exhibit a short-term efficiency, deteriorating and roughening as they age [5]. In addition, biocides and organic solvents present in protective layers have a negative impact in complete ecosystems where biofilms are treated [4]. With the pressure of stringent environmental regulations monotonically increasing, there is an urgent need for new environmentally friendly and sustainable microbial corrosion control strategies.

On the other hand, there is a phenomenon that, similar to microbial corrosion, includes metal ion release when a metallic material is in contact with other living systems, such as the one provided by human skin. Among metallic materials, Ni and its alloys are one of the most widely used in diverse domestic objects that come in contact with human skin. Ni has been chosen for most of these applications due to its mechanical properties, corrosion resistance, durability, appearance and availability. However, remarkable as these properties are, they do not seem to withstand direct interaction with human skin conditions. Release of Ni^2+^ ions from Ni metallic objects that come in direct contact with human skin has been related to a phenomenon known as the allergic contact hypersensitivity response to Ni [6,7]. This is one of the most frequent contact allergies produced by manmade products [7,8,9,10,11,12,13,14] and a major source of this Ni hypersensitivity.

Ni^2+^ is not only released from coins [15] but also from components of earrings, watches, buttons and dental and orthopedic implants, thus increasing the incidence of Ni^2+^-induced contact dermatitis [16,17,18]. Although Ni^2+^ ions have been reported to trigger an innate immune response in resident skin cells [19] they are not regarded as antigenic by themselves. The process seems to be a bit more complex: when ions attach to large proteins in the epidermis, our organism attempts to get rid of the nickel–protein complexes thus causing the emergence of the immune response, such as an inflammatory reaction on the skin [14]. When nickel-containing materials are in contact with the skin, metallic nickel can be solubilized by sweat, which is followed by the dermal absorption and skin penetration that leads to an enhanced allergic reaction to nickel [20]. This accelerated dissolution, promoted by immersion in sweat, is in fact the experimental condition used to test biocompatibility of Ni-containing materials, according to international regulations [21,22]. Reduction of nickel penetration through skin has been addressed by different topical agents [23,24], which in only a few cases have proven to be safe and effective [25,26]. Even rubber gloves are inefficient in preventing the contact with nickel, as metal ions penetrate through them [27].

Different nanoscale approaches have been used to produce certain degrees of control over contact allergy and microbial corrosion issues. For instance, the application of calcium phosphate and calcium carbonate nanoparticles to the skin seems to be effective in preventing the allergy towards Ni^2+^, by capturing nickel ions [28], which are subsequently removed from the skin. Although such approach seems to be effective, it raises concerns about the risks of absorption of nanoparticles through the skin [29,30]. Similarly, metal nanoparticle-based coatings have been also used for microbial corrosion control [31,32]. However, these coatings fall into the biocide category, exhibiting a strong bactericide effect coming from the release of metallic ions from the nanoparticles.

Recently our group and others have reported Cu microbial corrosion passivation using single-layer CVD graphene (SLG) grown on that metal [5,33,34]. Although SLG effectively protects copper, coupling between graphene and copper electronic states is very weak compared to electronic interfacial bonding in other graphene-metal systems, such as graphene on Ni or graphene on Ti/Au [35,36,37]. Electronic coupling becomes especially relevant when biological response of CVD graphene-coated metals is considered, due to the reported connection between electron transfer from these material systems to microorganisms and cell death [38]. When protection of metals (other than Cu) from biological interaction using graphene coatings is pursued, a different and interesting scenario is found. It is important to highlight that microbial corrosion cannot be completely linked to a single chemical reaction because it is influenced by the complex processes of microorganisms performing different electrochemical reactions and secreting proteins and metabolites that have effects over a material’s damage. Although corrosion-resisting properties of graphene coatings have been widely studied [39,40], evaluation of graphene performance under microbiologically-assisted corrosion conditions presents a different scenario, especially considering that graphene/metal system performance depends on graphene and underlying metal electronic interaction. From the biological point of view, graphene grown on Ni, Cu or any other metal or alloy behaves differently. In the case of Ni, for instance, graphene’s electronic structure is disturbed through a strong hybridization of the metal’s d-orbital and π graphene’s orbital forming a chemisorption interface [37]. In this scenario the strong coupling between Ni and graphene electronic states might play a major role in the biological response of the nanoscale-modified materials that needs to be addressed.

Motivated by the need of studies exploring the protection that graphene coatings offer to other relevant metals (other than copper), we present a study on the efficiency of graphene grown and transferred on Ni as an ionic barrier under two biological conditions, microbial corrosion and sweat immersion. Chemical vapor deposition (CVD) graphene, with surface areas in the centimeter square range, has been chosen as coating due to its versatility and lack of intrinsic cytotoxicity [41]. This condition is opposite to its close relative, graphene oxide (GO), formed by micro- or nanosized flakes of functionalized graphene in powder or solution, which has been shown to present an antibacterial activity [42].

In this work we explored the efficiency of as-grown few layer graphene (FLG) and transferred graphene onto Ni foils. We mainly focus on the way in which these nanostructured coatings modify interaction between: (1) Ni and bacteria (to test microbial corrosion passivation of such coatings) and (2) Ni and sweat (to evaluate Ni ion release from coated samples in conditions given by international test to quantify material biocompatibility).

## 2. Materials and Methods

Graphene grown on Ni and graphene transferred onto Ni surfaces were included in this study to establish a comparison between performances of these graphene coatings obtained through different methodologies. The performance of graphene transferred on Ni will allow a comparison between the same type of nanometric membrane but with different electronic interaction (hybridization) with metallic substrate; weak coupling for graphene transferred on Ni and strong for grown samples [35,36]. Graphene growth (for transferred samples) is performed through chemical vapor deposition (CVD) with methane as precursor using 25 µm thick copper foil (99.99% purity) as synthesis substrate. The CVD growth process was performed at 1000 °C under a methane-hydrogen flow rate of 20 sccm and 10 sccm respectively, as reported in Ref. [43,44]. Base pressure prior to carbon precursor gas entry to the furnace was 8.6 × 10^−5^ hPa whereas during graphene growth was kept at 1.2 × 10^−2^ hPa. A slight modification of this methodology is introduced by supplying this mixed flux in five steps of 20 min each. Between each step, the sample was held only under the hydrogen flux for 10 min to ensure a high coverage. The final methane step was followed by rapid cooling under a hydrogen-argon flux of 10 sccm and 20 sccm respectively. Similarly, few-layer graphene grown on nickel was obtained through methodology reported in ref. [45], using a 30 µm Ni foil substrate. Average coating thickness was 4-monolayer thickness according to Raman (see sample characterization section).

Single-layer graphene was transferred onto Ni substrate using the PMMA-assisted method [33]. For this, a layer of poly (methyl methacrylate) (PMMA) is spin-coated on the initial graphene grown on copper sample. Detachment of the PMMA/graphene layer is done by copper etching using 2.28 wt % ammonium persulfate aqueous solution. Graphene/PMMA layer is washed with deionized water, manually transferred onto the nickel surface and baked to improve graphene substrate adhesion. Finally, the PMMA layer is dissolved with acetone. The obtained graphene-coated Ni sample was used as substrate for a second, third and fourth transfer process, leading to four layers graphene transferred onto Ni (4MLG). The presence of PMMA residues is a drawback of this low-cost method and it is a critical aspect especially for electronic applications where PMMA scattering centers decrease carrier mobility [46]. For biological applications, such as those reported here, presence of PMMA residues does not affect either biocompatibility of the system [47] or bacterial adhesion to surface [41].

Corresponding Ni control substrates were prepared treating fresh Ni (NiF) foils (99.8%, Alfa Aesar, Tewksbury, MA, USA, 20 μm thickness) under the same temperature and hydrogen pressure conditions used for graphene growth but without the carbon precursor gas. Thermally treated Ni samples (control samples) will be called NiT. Samples of 1 cm^2^ surface area were used for all measurements.

A combination of scanning electron microscopy (SEM) (EVO MA-10, Carl Zeiss, Oberkochen, Germany), scanning tunneling microscopy (STM, UHV-VT, Omicron GmbH, Uppsala, Sweden) and X-ray photoelectron spectrometry (XPS; PerkinElmer PHI 1257, Perkin Elmer, Waltham, MA, USA, Al Kα source, 1486.6 eV) was used to characterize samples morphology and chemical environment. Prior to STM and XPS analysis, where ultra-high vacuum conditions are required (1 × 10^−10^ torr), samples were annealed at 100 °C for 20 min to remove all gases adsorbed on sample surface. Graphitic quality of as-grown FLG was investigated by MicroRaman measurements (inVia, Renishaw, Gloucestershire, UK, 532 nm laser) at ambient conditions at low laser power (<1 mW).

Surface hydrophobicity of coated and uncoated Ni samples was determined by contact angle measurements. For this characterization a drop of milliQ water (2 μL) was placed on the surface of coated and uncoated samples and images were immediately captured using a high-resolution camera. Bacterial hydrophobicity was measured following standard methods [48] with some modifications. A bacterial strain suspended in 40 mL of water, 0.5% NaCl and 2% NaCl were filtered on a micropore cellulose nitrate filter (pore size 0.45 μm, Sartorius^TM^ 1140647ACN, Sartorius Stedim Biotech, Goettingen, Germany) by filtration of the suspension using negative pressure. The filters with a bacteria film were dried at room temperature during 90 min in order to obtain a stable water contact angle measured by sessile drop method using 1 μL of distilled. The contact angle was measured based on image analysis [49] using the image processing software Image J (bundled with 64-bit Java 1.6.0_24, public domain) with the plug-in Drop Shape Analysis based on B-spline snakes algorithm developed by ref. [50].

To explore response of graphene coatings (FLG and 4MLG) to biological environments and their ability to prevent microbial corrosion of Ni, we used *Escherichia coli* MG1655 cultures, which have been reported to cause metal microbial corrosion [51,52]. Bacterial cultures were grown until prestationary growth phase in a low ionic strength medium that contained meat extract (5 g·L^−1^) and yeast extract (5 g·L^−1^). The bacterial cultures were concentrated by centrifugation (5000× *g*, 5 min), washed three times with Milli-Q water, and finally resuspended up to a turbidity of 3.0 at 600 nm. The turbidity of this stock dispersion is equivalent to a bacterial concentration of ~1 × 10^9^ CFU/mL^−1^ (Colony forming units per milliliter). This high bacterial concentration was chosen to ensure response of graphene coatings is robust enough, even for large number of bacteria. Milli-Q water was used as dispersant to avoid bacterial duplication and the forthcoming accumulation of mineral crystals that may interfere with the collection of microscopy images or cause unwanted chemical reactions with the samples. Nickel release from metallic samples exposed to bacteria was determined by Inductively Coupled Plasma (ICP) (Perkin Elmer Nexion 350D, Perkin Elmer, Waltham, MA, USA). Control samples (NiT) were exposed to Milli-Q water without bacteria. After 24 h, bacteria were recovered, poured into 2.5 mL of 15 μM EDTA (ethylenediaminetetraacetic acid) (Sigma Aldrich, Darmstadt, Germany) solution (pH 10) and centrifuged at 5000× *g* during 10 min. The supernatant was recovered, and the total Ni concentration was also quantified by ICP.

Cell viability (inverse to cell death) was monitored to evaluate the antibacterial activity of coated and uncoated Ni samples. Such antibacterial performance is a direct consequence of Ni ions release and, in our case, will be an indirect indicator of Ni microbial corrosion (in addition to previously mentioned ICP measurements). One volume (100 μL) of *E. coli* MG1655 stock dispersion was placed on each sample surface in order to obtain a final bacterial density of 60 μL/cm^2^. Sample + bacteria systems were incubated at 37 °C during 24 h in a humidity chamber to avoid evaporation. Once this incubation period completed, bacteria were recovered with 3 volumes of Milli-Q water using a standard micropipette. Cell viability at 0 and 24 h was determined using the microdot methodology [51]. Each experimental trial was conducted in triplicate. For SEM characterization, bacteria were fixed on samples with 3% (*v*/*v*) glutaraldehyde and dehydrated by washing with a graded ethanol series (from 10% to 100%), followed by critical-point drying and gold coating.

To study the response of graphene-coated Ni samples to sweat exposure immersion tests were performed for FLG, 4MLG and Ni samples. The artificial sweat solution was prepared similar to EN1811 [51], using 0.5 wt % sodium chloride, 0.1 wt % lactic acid, 0.1 wt % urea and normal ultrapure water (not aerated as described in the standard) [53,54]. The pH of the solution was adjusted to pH 6.5 using 1 wt % ammonia solution. Samples were exposed to artificial sweat for one week and then solution were recovered for ICP Ni ion concentration quantification.

## 3. Results and Discussion

**Figure 1:**
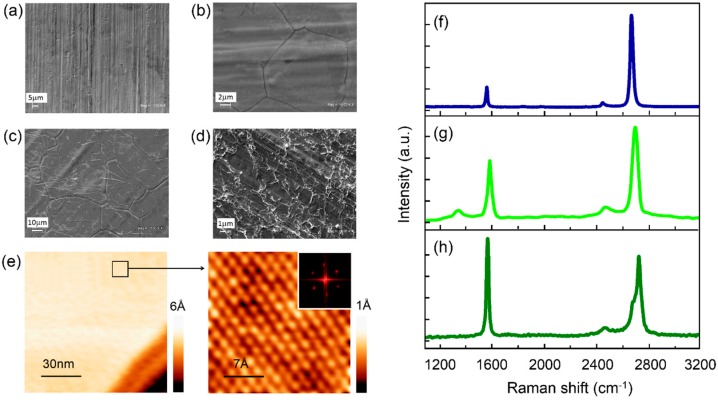
Structural and morphological characterization of samples. SEM images of (**a**) NiF; (**b**) NiT; (**c**) FLG on Ni; (**d**) 4MLG on Ni; (**e**) Large-scale STM topography (100 × 100 nm^2^) of few-layers graphene grown on Ni (I = 0.7 nA, V_BIAS_ = 0.1 V). High-magnification STM image (2.5 × 2.5 nm^2^) showing the triangular lattice atomic structure of few-layer graphene on Ni with lattice constant 2.4 Å, according to Fourier transform analysis (inset). Representative Raman spectra of SLG grown on Cu (for reference) (**f**), 4MLG tranferred on Ni (**g**) and FLG grown on Ni (**h**). Background caused by the luminescence of the copper was subtracted in the case of SLG grown and transferred on Cu.

Morphological characterization of samples prior to bacterial and sweat contact was obtained by SEM (Figure 1a–d). Fresh Ni samples (NiF) showed well-defined stripes across their surfaces (Figure 1a). In contrast, thermally treated Ni foils (Figure 1b) exhibited a smooth surface covered with deep grain boundaries and less pronounced stripe marks. Scanning electron micrographs of FLG grown on Ni showed graphene smooth areas bounded by wrinkles on top of nickel substrate (Figure 1c). Four layers of graphene transferred into Ni (4MLG) shows typical wrinkles produced during PMMA assisted transfer method (Figure 1d). This implies thickness spatial inhomogeneity of graphene coating across the sample surface, leading to thicker graphene regions where wrinkles are located. Reproducibility of transferred graphene coatings is lower than the obtained for grown graphene coatings, but in this case provide relevant information regarding a graphitic system where electronic coupling with metallic substrate is reduced.

Scanning tunneling microscopy in ultrahigh vacuum conditions was used to visualize FLG grown on Ni with nanoscale and atomic resolution (Figure 1e). Characteristic fingerprints of high-quality graphitic materials [55,56] were observed through atomic-resolved topographies. Zoom-in on a Ni terrace shows a triangular structure with 2.4 Å lattice distance (according to Fourier transform analysis).

To verify the graphitic quality of SLG, transferred graphene (4MLG) and FLG coatings, we performed microRaman measurements on these samples. Multiple areas of these samples were analyzed, and representative spectra are shown in Figure 1f–h. Single-layer graphene grown on Cu (Figure 1f) presents sharp G (1584 cm^−1^) and 2D (~2688 cm^−1^) bands, with a small G/2D (~0.23), consistent with literature [35]. Upshifting of 2D band with increasing the number of SLG transferred on Ni is observed for 4MLG (2D band at ~2698 cm^−1^). Raman spectra displayed typically sharp G (1584 cm^−1^) and broad 2D (~2701 cm^−1^) bands for FLG (Figure 1h), consistently with 4–5 layers of graphene, according to values reported in literature [56,57,58].

**Figure 2:**
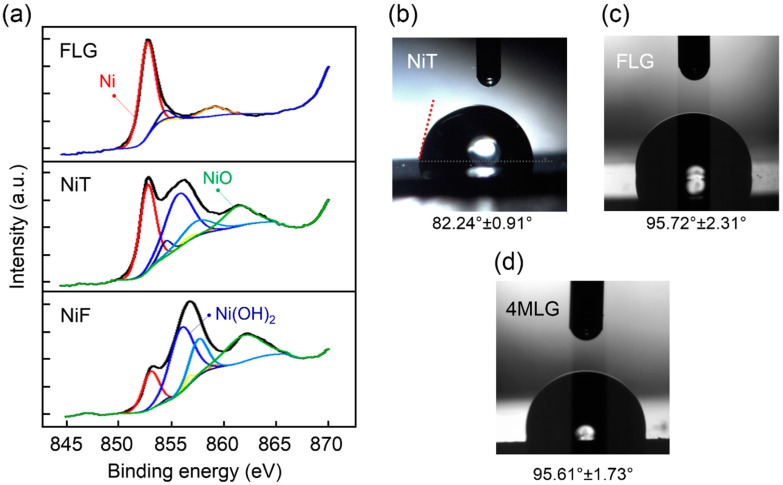
Surface composition and hidrophobicity of coated and uncoted Ni samples. (**a**) XPS Ni 2p_3/2_ spectra of coated and uncoated Ni, including peak fitting to identify Ni (red, yellow and orange), NiO (green) and Ni(OH)_2_ (blue) contributions. This allows the identification of FLG control sample (NiT). Images of contact angle measurements using Milli-Q water in contact with (**b**) NiT; (**c**) FLG and (**d**) 4MLG.

Surface composition information of Ni samples was obtained by means of XPS analysis (Figure 2a). In particular, we focused on the Ni 2p_3/2_ spectrum, which contains information on metallic Ni (peaks in red, orange and yellow), NiO (peak in green), and Ni(OH)_2_ (peak in blue) surface contents that are present when nickel is exposed to ambient conditions [59,60]. Stronger XPS metallic Ni peaks for FLG, NiT and NiF (in red) were found at 853.1 eV, 856.8 eV, and 859.1 eV, respectively. These measurements reveal that although Ni(OH)_2_ signals (in blue) are present for NiF and NiT samples (and to lesser extent for FLG), they are stronger for NiF. This is in agreement with previous reports where an initial Ni oxide growth in metallic Ni saturates at few layers after which oxygen in air leads to formation of Ni(OH)_2_ on the surface, that continue growing for longer exposure to atmospheric gas pressure [60]. Similar reports have shown the formation of ultrathin NiO coating when graphene is grown on Ni foams under low-pressure CVD conditions [61]. The resulting structure was a dual oxide configuration with Ni(OH)_2_ at the air-oxide interface and NiO between the metal and hydroxide layer. Nickel metallic signal is stronger for graphene-coated Ni than for bare Ni samples, regardless heat treatment. However, it is clear that NiT samples, due to oxide removal treatment, are similar to graphene-coated Ni in terms of metallic Ni composition, indicating that NiT is in fact the control sample for viability, microbial corrosion and sweat exposure measurements.

Contact angle measurements were performed to characterize surface hydrophobicity of coated and uncoated Ni samples (Figure 2b–d). Hydrophobic force is one of the most important properties involved in the bacterial adhesion process and is determined by physicochemical surface properties [62]. Bacteria are more prone to attach to the hydrophobic surfaces than hydrophilic surfaces [63], and hydrophobicity of the cell surface, such as that reported for *E. coli* [63], tends to increase adhesion [64]. In the case of nickel, there is a clear transition from hydrophilic surface (contact angle of ~82°) for NiT (Figure 2b) to a hydrophobic surface when nickel is covered by few-layer graphene (contact angle of ~96° for FLG, Figure 2c). An increase in the hydrophobicity has been also reported for graphene grown on Ni via SiC sublimation at 1620 °C [65]. Hydrophobic domains are connected to lower friction force, making the displacement of water molecules in wet environments an easy task for bacteria arriving to material’s surface that, in the end, favors their adhesion. A similar behavior regarding hydrophobicity is reported for CVD SLG grown on Cu [41]. According to our measurements surface hydrophobic property of FLG on Ni is not preserved for four layer of graphene transferred on Ni (Figure 2d with around 95°). These measurements show that bacterial adhesion is expected to be larger for FLG grown on Ni than for uncoated Ni.

**Figure 3:**
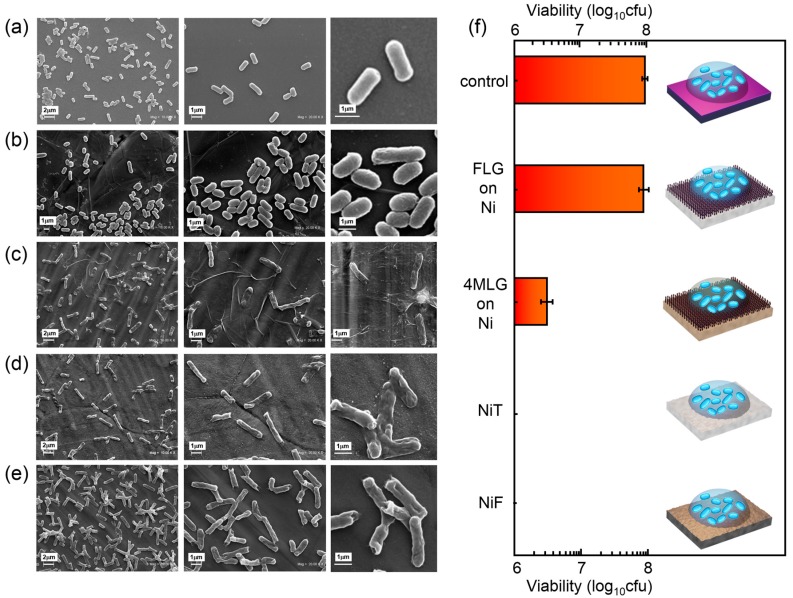
Biological interaction of graphene-coated Ni samples. SEM images of *E. coli* after 24 h incubation on: (**a**) SiO_2_; (**b**) FLG grown on Ni; (**c**) 4MLG on Ni; (**d**) NiT and (**e**) NiF. After incubation on NiT and NiF bacteria exhibit damaged membranes, irregular shapes, and rough surfaces, a clear sign of cell lysis. In contrast, intact bacteria were observed when incubated FLG grown on Ni foils. (**f**) Cell viability of *E. coli* MG1655 exposed to graphene-coated and uncoated Ni samples (and corresponding control SiO_2_) after 24 h. Fresh and treated Ni exhibit bactericide activity in contrast to FLG/Ni sample, confirming FLG coating substantially decreases the toxicity of Ni substrate to bacteria, which is connected to a reduced release of Ni ions.

In order to detect any interaction between bacteria and Ni that would lead to microbial corrosion, cell viability was measured. This approach is based in the fact that the main signature of microbial-induced corrosion is ions released from metal surface that, in turn causes, in the case of Ni ions, cell death [38,66]. Bacteria are used, for these purposes, as sensors of nickel ion release. Figure 3e summarizes cell viability results on *E. coli* after 24 h of incubation on NiT, NiF and graphene-coated Ni samples, together with morphology of bacteria after incubation (Figure 3a–d). Cell viability percentage (viability %) was calculated comparing CFU at 24 h and at t = 0. For our bacterial interaction measurements NiT was used as control sample of FLG due to the reduced Ni(OH)_2_ content in that sample compared to NiF (XPS results from Figure 2a).

SEM images of *E. coli* after 24 h incubation show that Ni-exposed bacteria (either NiT or NiF) exhibit a wide range of significant abnormalities (Figure 3d,e), such as a very rough surface or a hollow shape, suggesting that severe damage has occurred leading to the collapse of the cell structure. Such morphological features in bacterial cells are due to disruption of the cell wall due to ions influx, followed by leakage of the cell content [33]. Cell viability results for bacteria incubated on NiF and NiT confirmed these results, showing a zero viability value (Figure 3f), in agreement with the expected bactericide activity of Ni, connected to highly protein-reactive nature of their ions [66,67]. In contrast, smooth cell surface with regular shape is observed in the case of *E. coli* on FLG grown on Ni (Figure 3b), similarly to the control sample (SiO_2_) (Figure 3a). This is in agreement with our viability results, which show 100% and 95%, respectively (Figure 3f). For 4MLG on Ni a reduction from 10^8^ to 2 × 10^6^ CFU implies a resulting 2% viability. Transferred FLG and Ni is expected to present weak electronic coupling, which suggests that the observed bactericide effect is probably connected to graphene incomplete coverage (intrinsic to PMMA-assisted transfer process) and the consequent release of Ni ions.

Electron transfer from microbial membranes to graphene has been reported to produce a strong antibacterial effect in graphene grown on copper system [38], where the interaction between copper s electronic states with graphene π orbitals is weak [35]. If that were the case such electron-transfer bactericide effect would be amplified for graphene on nickel system, where a stronger chemical interaction results in a hybridization of the 3d valence-band states of Ni with the π-states of graphene [36]. However, similarity in the results for FLG and SiO_2_ (control) samples indicates that a connection between charge transfer from Ni to bacteria through graphene and their bactericide activity is less likely.

**Figure 4:**
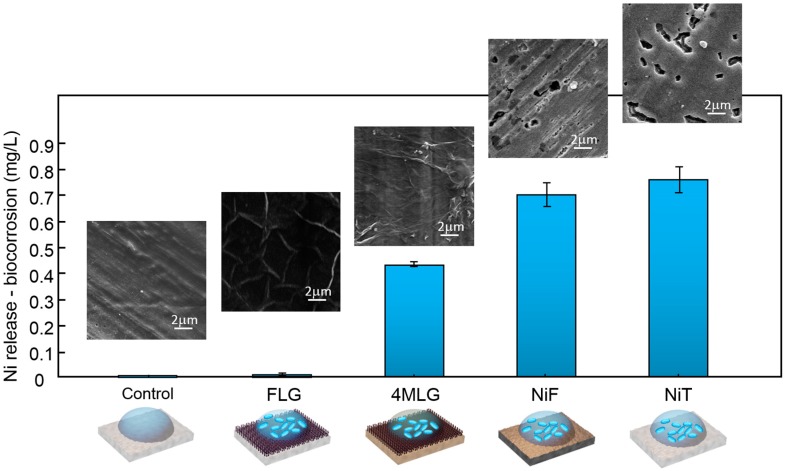
Ni ion release from graphene-coated and uncoated Ni samples. ICP measurements on coated and uncoated Ni samples after 24 h of bacteria contact showing Ni dissolution. Dissolved Ni for graphene-coated Ni samples in contact with bacteria (*E. coli*) is below technique’s detection limit, as well as control sample. NiT and NiF in contact with bacteria present high nickel ion release. SEM images show morphology of samples after bacterial exposure.

In parallel, nickel dissolution was studied by ICP to quantify microbial corrosion of graphene-coated and uncoated Ni samples due to contact with bacterial cultures. Figure 4 shows Ni ions released from samples exposed to bacteria for 24 h. NiT in contact with Milli-Q water without *E. coli* cells was chosen as control sample. Solutions with and without bacteria (control) do not contain salts than might affect electrochemical interaction with metal. Dissolved Ni from graphene-coated Ni samples (FLG) in contact with Milli-Q water with bacteria is close to the concentration obtained in control sample. Ni ions release is reduced up to 98.7% when using FLG coating (compare to NiT—control sample of FLG) and up to 46% when using transferred graphene (compare to NiF—control sample of 4MLG). These results demonstrate grown graphitic membranes efficiently protect underlying Ni from microbial corrosion due to bacterial interaction. In particular, Ni dissolution is found to be inversely related to cell viability (Figure 3f), which is in agreement with the proposed mechanism of Ni toxicity, which is thought to be influenced mostly by the influx of nickel ions into the cells. Metal dissolution (resulting from microbial corrosion process for instance) indirectly affects bacteria by incorporating ions into bacterial protein and thereby making the protein non-functional and/or resulting in malfunctioning [66,68]. Coated and uncoated samples were washed with de-ionized water to remove attached biofilm in order to visualize any possible damage at the samples surface. SEM images of NiT and NiF samples after interaction with microorganisms showed localized pit corrosion. Pits presented a non-equiaxed form with size comparable to bacteria dimensions. In contrast, FLG and 4MLG samples exhibited no visible damage, in agreement with ICP measurements.

**Figure 5:**
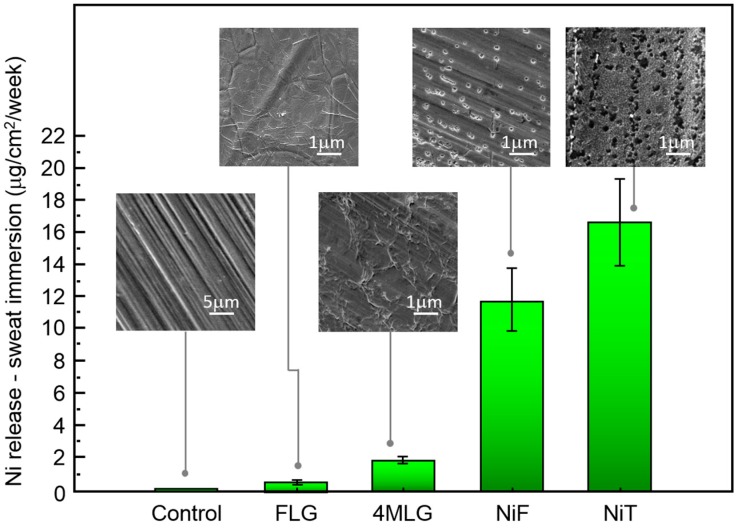
Ni ion release from graphene-coated samples exposed to artificial sweat. ICP measurements on grown and transferred graphene-coated and uncoated Ni samples after 1 week of sweat immersion showing Ni dissolution. Uncoated samples (NiT and NiF) in contact with sweat present highest Ni ion release. All samples where 1 cm^2^. SEM images show morphology of samples after sweat exposure.

To study the performance of graphitic coatings under Ni allergy conditions we performed ICP measurements on Ni dissolution for graphene-coated and uncoated Ni samples under sweat immersion conditions (source of acute Ni allergic contact hypersensitivity) (Figure 5). Nickel release limit, according the EN1811 standard developed by the European Parliament and Council (Directive 94/27/EC and amended in 2004/96/EC), is 0.5 µg Ni/cm^2^ per week for articles intended to come into direct and prolonged contact with skin [21,22]. Our results show that the release of Ni ions from uncoated samples (NiT) is 33 times higher than that limit (~16.5 µg/cm^2^/week) while for graphene-coated samples Ni ion release reaches a similar value (~0.6 µg/cm^2^/week).

Different coatings have been reported to be used to reduce Ni release from biomaterials in contact with artificial sweat [69,70]. In vivo experiments on human patients with metal allergy, using artificial sweet as eluent, have shown that when stainless steel disks are coated with a biocompatible surface coatings, such as TiN and ZrN, a reduction of around 70% in Ni release (compared to uncoated samples) can be reached after 5 days exposure [69]. Similarly, a 55% Ni release reduction has been found in NiT samples coated with a CaP nanometric layer after a week of exposure [70]. Remarkably, our results for 1 week sweat immersion (time defined by international regulation) indicate that FLG coatings are able to reduce up to 96% the release of Ni ions, far exceeding previously mentioned coatings. 4MLG coating reduced by 83% the amount of released Ni when compare with NiF, which is “control sample” for transferred graphene. The protection performance of transferred coatings under sweat immersion is higher than in the case of microbial corrosion conditions (Figure 4), which might be connected with different metal dissolution rates in both environments.

SEM micrographs of uncoated Ni samples after sweat exposure (insets in Figure 5) show a pit-like surface damage. Pit size was found to be inferior (and more symmetric in aspect) to the case of bacterial exposure. Coated samples morphology (FLG and 4MLG) is similar to samples before exposure, without any visible damage. SEM and ICP information confirms the ability of FLG coatings (and 4MLG to less extent) to reduce Ni ion release in Ni allergy test conditions. Differences between NiF and NiT dissolution can be related to composition of surface samples. According to XPS measurements NiF, previous to sweat exposure, displays a higher surface content of nickel hydroxide/oxide and lower content of metallic Ni than NiT. Such oxide porous layers tend to passivate the surface, not allowing a total surface protection but slowing the corrosion process [71]. Such partial protection might be connected to the observed low Ni^2+^ ion release for NiF, when compare with NiT.

Our results suggest that the observed effectiveness of graphene as a barrier against interaction between Ni and bacteria or sweat can be understood in terms of graphene permeability. Graphene’s p orbitals form a dense and delocalized cloud that blocks the gap within its aromatic rings [72], creating a repelling field, which does not allow molecules (or ions) to pass through. The reported graphene pore size is 64 pm [73], a value smaller than the effective ionic radii of Ni^2+^ (70 pm) [74], which are responsible for the antibacterial properties of Ni. In the case of FLG grown on Ni, the superposition of such impermeable effect for around 4 layers makes this barrier even more effective. Damage at graphene layer when transferred onto Ni (intrinsic to PMMA-assisted transfer process) leads to a lower efficiency barrier than in the case of grown graphene.

**Figure 6:**
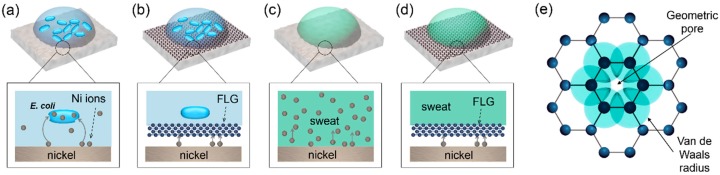
Diagram of FLG-coated Ni performance under biocorrosion and Ni hypersensitivity conditions inferred from our study. (**a**) Uncoated Ni samples (NiT or NiF) interacts with bacteria, releasing metal ions which lead to cell death; (**b**) For graphene-coated Ni, there is no interaction between bacteria and underlying substrate, leading to reduction of biocorrosion and, in consequence decrease of Ni bactericide activity; (**c**) Sweat, in contact with Ni (fresh or treated), boosts release of a high amount of Ni ions; (**d**) Graphene coating prevent Ni ions release in sweat which is related to Ni hypersensitivy in humans; (**e**) Geometric pore size of graphene determines a high impermeability to Ni ions.

Figure 6 summarizes the interaction between coated and uncoated Ni samples with bacteria and sweat. Figure 6a shows that a strong interaction between bacteria and uncoated Ni samples determines dissolution of Ni (biocorrosion), which ions penetrate microbial cells, inactivating their enzymes and causing death of microorganisms. When uncoated samples are exposed to sweat for a week (Figure 6c), a strong dissolution is observed, leading to a pit pattern on Ni surface. When the metallic substrates are covered by a graphitic membrane (grown or transferred) we observed a considerable reduction in Ni dissolution due to bacteria (biocorrosion) and sweat contact (Figure 6b,d respectively). The absence of electron-transfer bactericide effect in this highly hybridized system suggests this protection effect is presumably connected to impermeability of graphene membranes to Ni ions transfer (Figure 6e), similarly to the case of SLG on Cu, that suppress Cu^2+^ ions release from Cu foils in contact with bacteria [41]. The proposed protection mechanism is proven to be efficient even under more critical conditions, such as an increased bacterial adhesion to hydrophobic graphene/Ni surface.

## 4. Conclusions

In conclusion, we studied the performance of graphitic coatings as a protection barrier under microbial corrosion and Ni allergy conditions. From the microbial corrosion point of view, cell viability and Ni dissolution results indicate these nanostructured coatings effectively block interaction between bacteria and underlying metal. The absence of bactericide effect for this highly electronically hybridized system suggests that most of the protection from the observed protection from biological environment can be understood in terms of graphene impermeability to transfer Ni^2+^ ions, which is enhanced for a few layers of graphene.

This protective effect of impermeable graphene coatings is extended to the case of Ni allergy conditions. Although Ni dissolution does not remain below the value established by international Directive 94/27/EC in graphene-coated Ni samples, its proximity to this value confirms the great potential of graphitic coatings to prevent the significant amounts of nickel ions usually released from metallic devices, which may interact with the human body causing adverse health effects. The most effective way of preventing nickel allergy is the avoidance of exposure. The ideal case would be the complete ban of nickel use, which is irrelevant due the wide distribution of the metal worldwide. In this scenario the observed performance of graphitic coatings to prevent Ni ion release suggests a promising new venue to prevent allergy reactions. Further in vivo experiments are needed to evaluate biocompatibility of graphene-coated materials on humans and animals.
